# Rethinking social action through the info-ecological dimensions of two collaborative public health platforms: the people’s health movement and the citizen sense project platforms as examples of health-net-activism

**DOI:** 10.3389/fsoc.2025.1602858

**Published:** 2025-07-11

**Authors:** Silvia Surrenti, Massimo Di Felice

**Affiliations:** ^1^Department of Experimental and Clinical Medicine, University of Florence, Florence, Italy; ^2^Department of Communication and Arts, University of São Paulo, São Paulo, Brazil

**Keywords:** ecological health, trans-organic, infosphere, platform society, net-activism

## Abstract

**Introduction:**

The analysis of online platforms is usually restricted to their communicative properties, similar to analyzing digital infrastructures that facilitate interactions among users. However, the definition is missing a broader interpretation rather than tools or communicative channels. To review this instrumental vision, scholars in a variety of fields have begun to analyze platforms from a multidisciplinary perspective as technical, economic, and sociocultural ecosystems that characterize the structure of contemporary society.

**Methods:**

In this article, we adopt an info-ecological approach to the processes of platformization through a qualitative analysis of two platforms dedicated to health and quality of life. The infoecological approach suggests a new living condition that promotes the emerging computational ecologies composed of a web of people, data, algorithms, biodiversity, information, cities, viruses, and so forth, supporting a more-than-human common experience.

**Results and discussion:**

The purpose is to examine how the heterogeneity of platform ecosystems (human and non-human) have been generating a cultural shift. That is to say, a-more-than-human interconnected and trans-organic network of networks that in our perspective also represent what we have called a new type of health-net-activism and digital citizenship.

## 1 Introduction

The article aims to analyze the ongoing changes in the fields of social and health engagement due to the diffusion of digital platforms, from what we have called an info-ecological perspective. The focus is on social practices implemented by platforms to exchange and produce data with/from people and the environment, leading to new forms of social activism we have called net-activism.

The analysis begins with changes brought about by the digitalization, particularly the rise of the platform society (van Dijck et al., 2018), which has resulted in the diffusion of hybrid interconnected agencies that are transforming traditional social action into more-than-human ecosystems (human, environment, data). This is a social act that is not only human-centered and transitive (inward-outward), but also connective and networked.

We will look at two collaborative “public health” platforms, the People’s Health Movement Platform (PHM) and the Citizen Sense Project Platform (CSP). The PHM platform is a global network of grassroots health activists, civil society organizations, and academic institutions working to create a more equal and healthier world, whereas the CSP platform—always a network of different stakeholders, as any platform structure implies (van Dijck et al., 2021)—involves people in sensing technology to co-create environmental data for air quality and public health.

We refer to such platforms as info-ecologies of net-activism, which have been described as expressing the trans-organic (biological and digital) components of contemporary living, as they are not purely human, environmental, artificial, or digital.

Floridi (2016) coined the term infosphere to define the informational space of the digital era which encompasses all aspects of life and presents humanity with new societal challenges. The infosphere is the space in which humanity spends an increasing amount of time and engages in an increasing number of activities. Even the physical space is impacted by the infosphere where algorithms are used by wearable sensors, digital platforms, social media, among others, to gather and produce data for monitoring, controlling, and enhancing human existence. Similarly, Van Dijck et al. (2018) argue that platforms, rather than producing a revolution, are converging with offline organizations’ institutions and practices, resulting in a new hybrid society known as *platform society*, which is home to a new hybrid “participatory culture.”

In this sense, PHM and CSP platforms have been credited with fostering a new type of health activism inspired by digitalization, which we call “net-activism” in general and “health-net-activism” specifically.

Van Dijck et al. (2018) have emphasized the concept of platforms infrastructures for online services as << complex interaction between users, practices, technologies, and business models - a combination of human and nonhuman actors> > (p. 2); which is, platforms as living environments generating data and new forms of social participation. In line with these authors, our perspective is based on the understanding that networked digital technologies, rather than being simply tools, instruments, or support to be used, are primarily “environmental forces” that alter who we are, how we socialize, and how we interact with reality (Di Felice, 2017; Floridi, 2016; van Dijck et al., 2018).

Digital platforms as new living ecologies extends social action into a connective world that is algorithmically organized by data, allowing people for co-creation and interactions of contents beyond simply searching for information (Vicari and Cappai, 2016).

Just to mention a few examples in the health field, digital platforms and social networks such as *PatientsLikeMe* have provided people with emotional support and mental confidence, while also exchanging information about one’s disease for medical research (Tempini, 2015; Lupton, 2013). Smartphone applications have engaged people in socially and politically relevant health issues by tracking infectious diseases and monitoring chronic disorders (Hernández-Orallo et al., 2020; Meskó et al., 2017). Wearable devices, such as Google Glass and Fitbit, that record biometric information (e.g., body temperature, heart rate, blood glucose, food calories, etc.) have increased physical activity and shifted people’s attention to preventive lifestyles (Henwood and Marent, 2019), with datafication becoming a part of people’s identity construction (Lupton, 2020).

It is known that digital technologies may also provide negative effects on human psychology, such as psychological distress, fake news exposure, personal information exploitation, and apathy (Dienlin and Johannes, 2020). However, in a health engagement perspective, digital devices and platforms have been largely utilized to increase well-being and living, as well as new forms of health activism set through the use of data as people and sensors may generate content (Spanakis et al., 2016; Eysenbach, 2008; Petersen et al., 2018; Saukko, 2018). It is no coincidence that digital health is linked to new models of healthcare that are both predictive and personalized, as well as peer-to-peer and lay-expert interactions (Lupton, 2021; Metha, 2011) which are empowering patients and citizens as participatory medicine and citizen science grow (Hood and Friend, 2011; Ruckenstein and Pantzar 2017; Erikainen et al., 2019).

As we will see through the case studies, platforms are promoting a connective social action that is not only a human transitive experience (inward-outward) but a connective one, in which sensitive technologies (such as wearables) generating data are metaphorically making non-humans “speak.” For instance, through the making of data on the quality of the air we breathe or the soil we live in, as is the case in one of the two platforms analyzed.

The goal is to emphasize the rise of new connective health ecologies where organic and inorganic factors meet and in which data, information and people interact to create communicative ecosystems of health-net-activism; which is rethinking social action as a connective and trans-organic experience.

The analysis develops as follows: first, reviewing the idea of platform society; second, applying the idea of platform society to the health and wellbeing domains; third, presenting an analytical framework as well as a platform typology, and finally, analyzing the PHM and PSC collaborative “public health” platforms as examples of new forms of digital activism, the health-net-activism.

## 2 Platformization of society, platformization of health

### 2.1 Platformization of society beyond the idea of “technology as an instrument”

In the Western world, technology is commonly interpreted as instrumental, as a tool which serves human activities, representing a human body and cognitive extension to better deploy the surrounding environment (Puech, 2008). The concept of “technology as an instrument” also applies to digital platforms, which have been often relegated to the role of practical objects or supports of communication.

Software studies in business and scholarly communities, critical and political economy, and cultural software studies have all supported the idea that technology is only an instrument (Abdelkafi et al., 2019; Baldwin and von Hippel, 2011; Yoo et al., 2010). Helmond’s (2015) definition of platformization, which describes the penetration of platform extensions into the web as well as the process by which third parties make their data platform ready, is a widely held viewpoint in computational studies.

Also engineering design has theorized platforms as modular technological supports or architectures (Gawer, 2014). Similarly, economic theory has conceptualized platforms as utilities for double-sided markets, complementor and end-user (Rochet and Tirole, 2003). In other words, platforms as interfaces allowing data flows with third parties, and therefore technological instruments for others to build on.

On the contrary, political and cultural insights are broadly interested in emphasizing the fact that platformization processes empower people by offering an opportunity to communicate in a participatory way, shaping new types of networked sociality (Gerlitz and Helmond, 2013). Consider the extreme case of making platforms necessary social environments at the onset of the Covid-19 pandemic, and during the consequent lockdown of cities around the globe. At that moment, hundreds of millions of users became even more dependent on platforms and apps as digital environments to work, socialize, learn, and be entertained (Fox, 2022).

Following this line, in the sociology of media, van Dijck et al. (2018) claim that online platforms are neither an exclusive economic phenomenon nor a technological construct that facilitate various kinds of user interactions, but rather a dynamic and hybrid process, that is, an active part of an ecosystem which has penetrated the heart of societies affecting institutions, economic transactions, social and cultural practices. To emphasize the inextricable relation between online platforms and societal structure, van Dijck et al. (2018) employ the term *platform society* meaning that platforms, rather than reflecting the social, actually produce the social structures we live in today.

Similarly, Poell et al. (2019) describe platformization as the penetration of the infrastructures, economic processes, and governmental frameworks of platforms in various societal sectors and spheres of life (journalism, transportation, entertainment, education, finance, and health care), as well as the reorganization of cultural practices and imaginations around these platforms. For all these reasons, Gillespie (2010), Poell et al. (2019), and van Dijck et al. (2018) argue that the term platformatization needs to transcend its computational or double-sided business meaning (end-users and complementors), resulting in a complicated configuration of platforms that have been depicted as ecosystems of connective media which are gathering heterogenous players and interests, such as government interests, markets and populations (van Dijck et al., 2021), but yet far beyond the idea of platforms as technological instruments.

Various scholars have discussed the criticalities of platformization, such as digital divides (bias) due to data ownership or control over digital infrastructures, as they might lead to asymmetries among stakeholders (van Dijck et al., 2018; Rubeis, 2023; Zuboff, 2015). According to van Dijck et al. (2018) health platforms produce norms and values that bring criticalities such as the dispute concerning private gain versus public benefit. An example from the health sector is the *23andMe* platform, which allows users to sequence their DNA through learning about their genome. While users claim they rely on this type of platform to estimate the potential risks on developing specific diseases (private goal), the platform experts declare they are pursuing the goal of educating people to manage their own health by bringing genetic science closer, aiming towards better public health (public benefit).

According to software-code scholar Accoto (2017), the ethical knot described by van Dijck et al. (2018), of private against public interests, becomes intertwined one we think of platforms as operating systems at the junction of free markets and hierarchies. This is why systems that facilitate interactions and partnerships between many stakeholders are placed together in the framework of a platform ecology.

In the study area of business economy, various authors have described private and public divide in digital platforms from an ecological perspective (Calabrese et al., 2021; Srnicek, 2016). This perspective is interesting because it acknowledges a distinctive characteristic of platforms; that is, the power to subvert business status quo and the commercial value chain, as that of traditional market structure, by combining various functions: technologies, players, interests, and goals.

Srnicek’s (2016) concept of “platform capitalism” is crucial for the success of these multi-stakeholder platform ecosystems. According to this author, one critical component of the platform society is its monetization through the extraction and marketing of user information. Platform capitalism collects vast amounts of data from people’s interactions, which is then sold without direct recompense to users. Indeed, Srnicek (2016) defines platform capitalism as a new type of economy based on numerous network effects and the exploitation of workers who are not subject to labor laws since they are classified as independent contractors. Bratton (2015) explains such a shift in economy by the fact that platforms are “governing architectures” that combines the logics of both states and markets. In the end, the platformization of society is a complex process that is reconfiguring how power is exercised and how we interact with the world.

#### 2.1.1 The info-ecologies of health platformization and the rise of new forms of “social” activism

In this complex context, health platform ecosystems show new options for citizens and patients to manage their health and trace their identities (Matwyshyn, 2019), as digital health has been promoted as an empowering expression of participatory medicine and citizen science (Erikainen et al., 2019; Lupton, 2021; Metha, 2011).

The Web 2.0 system of communication (reading/writing) has made interactivity accessible and enabled patients and citizens to access and produce information sources to share experiences with peers, as well as obtaining some expertise regarding their condition to proactively manage their health (Petersen et al., 2018; Saukko, 2018).

The user-generated contents of digital platforms may be addressed to produce data to advocate changes in health-related policies or to get emotional support (Jacobs et al., 2017). Crowd-science – or people’s direct involvement in science, such as data collection – has indeed proven to be a valuable opportunity to improve medical research or, in the case of rare diseases, to generate more inclusive access to treatments and care than in the past, as well as new types of health social activism and public health control (Lupton, 2013; Chen and Wang, 2021; Di Felice and Surrenti, 2022). For instance, in cases of chronic diseases digital health elevates the patient’s confidence in his or her own care management abilities (Eysenbach, 2008).

Deborah Lupton (2021), who investigates how applications and data matter in everyday life, takes a more-than-human perspective to digital platforms, presenting them as dynamic assemblages of humans and data agencies forming connections that influence states of wellbeing. In her perspective, users appreciate digital devices designed to monitor specific aspects of health associated to better control of body functions or to prevent and predict specific disease predispositions.

For all these reasons, some scholars have regarded platformization positively, as an affordance for a new type of digital activism, particularly in the health sector (Vicari and Cappai, 2016; Petersen et al., 2018; Petrakaki et al., 2021). Petersen et al. (2018) argue that patients’ increasing reliance on digital media has become important to new power analytics, linking disease-specific groups’ interests with those of research and commerce. According to these authors, digital media alters not just people’s access to information, but also their ability to create and use information, such as using websites to campaign for medical treatments and healthcare rights or to influence science agendas.

Petrakaki et al. (2021) see digital platforms as productive of citizenship, fostering expressions of compassion, belonging, and demands for change in healthcare, while also empowering individuals to care for themselves and others.

Vicari and Cappai (2016) demonstrate that digital media offer people tailored paths to public health engagement, where experienced knowledge and medical authority are equally valued. According to these authors, digital activism advocates for the inclusion of non-scientific and non-governmental views in the management of public health, expanding health discourse practices and mobilizing and connecting different publics in ecosystems of social activism. That is, the development of bottom-up sharing and co-production of knowledge.

In this context, Petersen et al. (2018) argue that digital media and platforms provide a new living environment of action and information for citizens seeking to actively control their health. The authors discuss the emergence of a new type of health activism that replaces bio-sociality with bio-digital-citizenship. Digital health activism is here described as people that associate the fight for their rights with the use of data to influence social policies and receive the best care, as well as to contribute to research, monitor public health, or monitor the urban quality of life.

Indeed, platformization of health may combine lay experience and credentialed expert information other than in a cooperative way, even in a challenging setting (Labonté, 2013; Petersen et al., 2018). As previously underlined, there are other intermediate actors in these new health-ill ecosystems, apart from citizens and patients, which could act for-profit in medical research industries, and for data production and commercialization of information as well (Srnicek, 2016). For instance, consumer technology corporations such as Apple and Google are becoming important hubs at the passage points of data-intensive precision medicine (Accoto, 2017).

In this respect, deepening the discussion, Sharon and Lucivero (2019) claim that the technological tools needed to capture and organize data - from wearables and smart phones to cloud platforms and machine learning - rely on agencies that lie beyond the scope of traditional medical scientists, such as communication technology specialists. In this context, new stakeholders, human and data-driven agencies, enrich the medical scene. What is important, is that these new kinds of people-data connected experiences come to light outside of the traditional institutional paradigm, as people collaborate to produce them through the daily use of wearable devices and the entire platformization ecosystem (Sharon, 2018).

Similarly, Meskó et al. (2017) and Topol (2012) see digital platforms as the key to breaking down the status quo of traditional care models, which are usually limited to the one-on-one, asymmetrical relationship between doctors and patients in a hospital or outpatient setting. Meskó et al. (2017) assert that it is possible to trace the cultural shift within the status quo of care and health in a new phenomenon known as digital health, defined as the cultural transformation of how disruptive technologies that provide data accessible to both caregivers and patients lead to an equal level doctor-patient relationship with shared decision-making and the democratization of care. Given the potential of the new platform economy - the ability to network, innovate, and co-create value by collaborating with data - it is possible to envision a generation of new health info-ecologies in which multiple stakeholders are involved and data and information play a crucial connective role.

In our opinion, the leading role that doctors and patients, as well as all the other stakeholders, play in the digital realm, together with information and data spread, is what create multiple connected info-ecosystems, which are the new living conditions of caring, prevention, and public health.

As we will see through the case-studies, digital health plays a central role in health innovation, as it facilitates citizen/patients’ participation in the process of caring and in that of public health, linking different stakeholders, such as institutions, private corporations and civil society, and even environmental features, not in a hierarchical way, but in new forms of net-activism.

We refer to this combination of sociocultural shifts as info-ecologies of net-activism (digital ecosystems). People’s health and care pathways are becoming increasingly similar to collaborative informational ecologies organized around human and non-human entities (e.g., technology, data, algorithms, viruses, bacteria). This contributes to a new identity for the subject in general, and the patient in particular, similar to the concept of info-vidual (Di Felice, 2019), defined as the inseparable whole of the physical and digital person, the former presented in organic form, the latter composed of a set of online data and digital profiles.

Such new digital info-ecological ecosystems are home to new forms of social activism we have called net-activism, which expresses itself not only through protest or political movements, but through the constitution of collaborative efficient data-networks, in which new net-social actions articulate their architectures through content production and its distribution.

There are some common characteristics that mark the quality of net-activism that we will present in the next paragraph, and in which we suggest an interpretative scheme of two platforms for public health net-activism.

## 3 Materials and methods: the theoretical framework of the info-ecological approach

We have taken an info-ecological approach to address platforms and practices of platformization. The approach we have adopted is situated at the nexus between science and technology studies and sociology of health. It originates from the evolution of the web that presents ecological potentials due to its connective and participatory nature.

The info-ecological scale of interpretation is the result of the fact that today we inhabit a new social, comprised not only of physical realities, but also of info-realities (Di Felice and Surrenti, 2022). The new social is composed of data, people and surfaces that are equally physical, virtual and connected, communicating and interacting with each other through the process of digitalization.

In line with van Dijck et al. (2018), the info-ecological approach describes a new type of communicative architecture for platforms that are interpreted as new living conditions. It supports the emergence of a computational ecology, which consists of a web of people, data, algorithms, information, cities, viruses, and so on, changing the definition of social as limited to only human agency.

The info-ecological approach is based on the fact that the co-evolution of everyday life is a more-than-social common (Haraway, 2012; Morton, 2017), and that, like any biological system, alters the agency of all members in response to the presence of other co-inhabitants (Tansley, 1920) and their reliance on information and data. It is a novel morphology of the social that is connected and hybrid since it is both organic (people, territories) and inorganic (data and information), which we refer to as trans-organic.

Similarly, platforms represent a new ecological living condition shaped by the many different communities of users, stakeholders, objects, territories, data and algorithms: a trans-organic network of entities composing and expressing a different complexity than the system of social relations proposed by social science, which reduces social dynamics to a set of relations between human subjects and institutions (Di Felice, 2019).

This analytical approach has been applied to two collaborative “public health” platforms: the People’s Health Movement Platform - Health for All Now [PHMP], which promotes health and network activism; and the Citizen Sense Project Platform [CSPP], which investigates urban environmental quality and well-being using sensing technologies and citizen participation to promote public health.

These two platforms have been regarded as examples of new communicative architectures we have called info-ecologies of participatory public health and care, as well as a new form of social engagement we have called net-activism, which informs society and its structure, transforming the traditional social act into one of more-than-human connectivity.

We have eschewed one-sided, monolithic understandings of platform dominance in favor of a theory of connected and interrelated platform dynamics that is relational and ecological. As van Dijck and Poell (2016) emphasized, giving the complex nature of the platform society, it is essential to combine several theoretical viewpoints and approaches to trace the diverse agencies of the current platform ecosystem.

We used a hybrid approach of qualitative inner analyses of the two platforms, PHMP and CSPP, that is descriptive and narrative of the online context, influenced by grounded theory, as well as online ethnography observation, to suggest a descriptive manner of mapping the two platforms. At the same time, we identified unique aspects which mark the nature of net-activism.

Grounded theory is the process of constructing hypotheses and theories by collecting and evaluating data from the study area (Chun Tie et al., 2019). We then coupled this methodology with digital ethnography narrative (Forberg and Schilt, 2023). Researchers face a cultural shift in which online lives and activities are becoming integrated into people’s daily lives, as social activity that is integrated into platforms (Bluteau, 2019). Researchers must also be immersed in data to observe the new hybrid social we are all living in. We evaluated online material using grounded theory and digital ethnography to root an ensemble of critical thinking on the new social action in a culture of info-connections.

The general aim of the study is to look at how digital health platforms suggest a certain idea of social action, like changing the health system by working together with people, data, algorithms, and territories, going far beyond just the simple doctor-patient relationship or the institutional healthcare setting. Platformization of society, and more broadly digitalization, is essentially creating a new version of the social as a result of digital networked ecosystems that, as previously stated, are not only informational suppliers but also co-producers of social reality (van Dijck et al., 2018).

We described the collaborative health and preventive care platforms using the interpretative framework provided below, taking into consideration the following dimensions:

1. Form and purpose of the platform.
2. What the platform allows or does not allow.
3. The degrees of interaction.
4. Patient or Citizen Engagement.

We then engaged the descriptive level in what we believe are some of the common characteristics that identify net-activism:

1. It is ecologically organized around people, data, and environmental features (ecosystem).
2. This is a trans-organic action that involves both organic (human, environment, animals, etc) and inorganic agencies (data and algorithms). It makes non-humans participate through sensors which collect data and making non-humans “speak” through informatization.
3. It represents multiple connected localities and stakeholders.
4. It represents an action that is not linear (inward to outward), but rather interrelated and connected. There is no hierarchy.
5. It is not the expression of a single human subject-actor, or of a single social movement, but the result of the synergy of multiple humans and non-human agencies.
6. Net-activism, as its platformization, redefines each entity no longer as an autonomous reality, but as part of a relational form that it acquires its specific condition only through different interactions and connections.

The specific aim is to describe the moving from the act of “communicating through” (the transitive way – from A to B in which technology is still an instrument) to “communicating within” (the networked action in which technology is part of the social structure). It is the process of communicating in a network of networks creating a new living condition of info-ecological space and trans-organic interactions (organic and inorganic), which is giving rise to new forms of health-net-activism, whose features we will present in the discussion paragraph.

## 4 Results: framing the research with platform description

We investigated the People’s Health Movement Platform (PHMP) and the Citizen Sense Project Platform (CSPP) because they both represent new ways for people to participate in a new social common in which the social act evolves from a transitive experience to a connective one (the net-activism), which is a trans-organic dimension that is not limited to humans but also to data and territories (such as air quality and land pollutants, as we will see in the CSPP case) connected to the same ecological system. Such a process is taking place in the name of platform affordances in which agencies, or performative abilities, are distributed across numerous stakeholders, both human and non-human, such as data and information, as well as people, territories and biodiversity.

### 4.1 People’s health movement platform—health for all now [PHMP]

We refer to such a platform as a new living environment for health promotion and as a bottom-up and hybrid type of net-activism. We shall read through its description.

#### 4.1.1 Form and purpose of the platform

PHM platform symbolizes a data-people care ecology, a network of algorithms, health activists, civil society organizations, and academic institutions from all over the world, notably from low and middle-income nations. It is a trans-organic experience that combines humans (organic) and a data (inorganic) ecology.

PHMP describes itself as a grassroots movement that operates in over 70 countries with the purpose of achieving universal access to health care. The platform works on a variety of programs and activities and is dedicated to providing comprehensive primary health care that addresses the social, environmental, and economic determinants of health. In this sense, the platform is both locally and globally located representing multiple stakeholders.

The Charter for the Health of Peoples encapsulates the PHMP’s fundamental ideas, which highlight civil society’s active participation in health decision-making processes. One of the platform’s objectives is to encourage group participation in the development, implementation, and evaluation of all health and social policies and initiatives. The Chart, which is the movement’s strategic guide, and the platform ecosystem, are both advocacy environments for action of health as a fundamental right. According to the platform, equity, sustainable development, and peace are essential for a better society in which everyone may live a healthy lifestyle. Indeed, as previously said, net-activism is the product of synergy across several agencies rather than home for a single subject.

As a network organization that calls to action, PHMP promotes communication and connects the various stakeholders of the social movement. This type of policy collaboration has resulted in a steady stream of articles, submissions, and pronouncements that are the info-ecologies of the movement. A thematic example of a more-than-human social is the section dedicated to the renewal campaign using data on gender justice and health, nutrition and food sovereignty, equitable health systems, and environment and ecosystem. The platform gives birth to a new social common made out of people, data, and territories consisting of numerous layers and components.

#### 4.1.2 What the platform allows or does not allow to do

The platform provides ample space for its members’ educational activities and engagement with global health organizations to guarantee fair access to care. Over the last year, the movement has launched an initiative to ensure equal access to crucial technology in the context of Covid-19. Among the training activities, the course *The Struggle for Health*, part of the International People’s Health University Program, offers training that differs significantly from traditional academic courses.

The course is described as a series of health activist trainings free to all citizens. It is emphasized that the university is no longer the only venue where information is structured, but rather that communities, in accordance with their digital surroundings and as a result of offline encounters, have evolved into the new gymnasium of knowledge. Teaching activities bring together a varied collection of faculty members (students, professionals, practitioners, and researchers) from over 20 nations and professional areas (ranging from health professions to social sciences). The training activities take place horizontally, fostering contact between instructors and learners, also known as organizers and enrollees, in a working atmosphere that encourages knowledge sharing. In this sense, net-activism has no hierarchies and it makes of education a new means of struggle for health.

The platform promotes itself as a new public environment where people and organizations from all around the world may be mobilized to support innovative health initiatives and social policies. One of the movement’s key objectives is to urge people to seek alternative solutions to local health issues. This is the case for a type of net-activism in which global meets local.

As in any ecosystem, the PHMP is a network of networks which serves as a catalyst for sharing experiences and developing new resources, such as studying and analyzing international health association documents, building networks among organizations and individuals to advance health campaigns at the international and local levels, and advocating for actions at international organizations.

#### 4.1.3 The degrees of interaction

The platform describes itself as a “network of networks” in which links are strengthened both locally and internationally, with each node connecting to many networks, and what we have underlined as the distributed and connected nature of platforms. Although the documents make no mention of it, the network’s very existence facilitates these connections by linking members and data. In the experience section, the movement provides mixed-mode (online and offline) training, such as the Gender, Justice, and Health course.

#### 4.1.4 Patient or citizen engagement

According to the platform, technologies are intrinsically linked to the health paradigm, influencing our conceptions of illness and treatment. To improve the approach to health, both within institutions and through bottom-up experiences, it is critical to explore whether medical technique can be separated from managing health disparities. Today, digital technologies have converted into new living environments, and this group’s lobbying appears to be inextricably linked to the growth of digital health ecosystems. Citizens’ engagement in this scenario is cognitive, emotional, and behavioral. The platform’s distinguishing features include trainings and advocacy actions which are part of the net-activism paradigm.

### 4.2 The citizen sense project platform—investigating environmental sensing technologies and citizen engagement

We refer to such a platform as one that allows people to co-create and participate in environmental data that benefits public health.

#### 4.2.1 Form and purpose of the platform

The platform houses the Citizen Sense project. The project’s purpose is to investigate the relationship between environmental sensing technology, behaviors, and citizen participation. The major purpose of such a citizen-sensing project is to democratize the collecting and utilization of environmental sensor data in order to promote wider citizen engagement in environmental issues for bottom-up public health participation. In this sense, the platform is ecologically organized around people, data, territories, and environmental features, in accordance with net-activism principles.

A second purpose, in addition to gathering crowd-sourced data sets, is to assess the viability of promoting new forms of environmental consciousness and activity. The research project is founded on the fact that tools for monitoring and sensing environments have migrated to everyday participatory applications, such as smartphones and networked devices, capable of engaging with new modes of environmental observation and data collection.

The platform as ecology seeks to broaden and challenge the potential for democratizing environmental initiatives through citizen sensing techniques, including significant fieldwork and the usage of sensing applications. Citizens net-act through programs such as pollution sensing, which identifies environmental disturbances such as air and water pollution; urban sensing, which employs sensor technology to promote urban sustainability; and wild sensing, which maps and records flora and fauna activities. In this sense, net-activism links local problems to global issues, such as climate change.

The platform is made up of multiple layers that together with data build a more-than-human social ecosystem. The first layer is the home environment, which showcases a citizen-sense and data collection project that promotes public participation and science democratization. For example, how persons who utilize smart phones or networked devices can participate in environmental observations and data collecting, or how the citizen sense project works with specific social communities to monitor air quality levels.

A second layer is dedicated to initiatives that take into consideration human technology entanglements as well as data collected by sensing projects. A third level focuses on goods that can let people become involved in the sensing project (kits, events, walks, laboratories, and movies), while a fourth layer collects Covid pandemic data in specific geographic places. In all circumstances, the study seeks to monitor air quality and assess contaminants exposure for participatory public health. The project’s goals are to develop an air monitoring kit that serves as a citizen-sensing facility, as well as to investigate the usage of digital Wi-Fi environmental sensors to monitor and report on environmental pollutants.

#### 4.2.2 What the platform allows or does not allow

The platform allows conducting environmental research using sensing technologies for citizen engagement. The platform is dedicated to sensitive research including citizen data. As stated on the platform, the goal is not only to engage citizens in collecting environmental data, but also to introduce new practices of environmental care.

#### 4.2.3 The degrees of interaction

The civic engagement platform offers a variety of sensing devices developed by various collaborators as a result of citizen science research, as well as seminars, walks, and workshops where sensing methods are evaluated. It also allows residents to collaborate on data collection projects. Among them are the airkit, a citizen-sensing toolkit for monitoring air quality; and the phyto-sensor toolkit, which works with plants that are sensitive to air pollution with the goal of creating air-quality gardens and monitoring air quality using inexpensive sensors. The various kits available enable people to take part in one-of-a-kind projects, such as a data-collection project with neighbors to create an air pollution monitoring kit. Alternatively, a citizen initiative with participants to test Airkit technology during the Covid pandemic.

#### 4.2.4 Patient or citizen engagement

The platform supports cognitive, emotional and practical engagement with data by allowing users to approach and co-create knowledge. It is a sort of health literacy action that is gaining popularity due to the publication of research data and possibilities to learn new skills. The platform promotes emotional and active participation by aggregating data acquired by users using their own sensors, thereby democratizing biosphere data and increasing citizens’ environmental responsibility.

## 5 Discussion: rethinking social action through info-health, net-activism, and trans-organicity

PHM and CSP platforms are part of a collaborative info-health ecosystem as well as a network of social digital agencies we have called net-activism. Both case studies demonstrate how social action (and education in PHMP case) is being rethought through the relationship with information and data on the one hand, and the connecting network with people and non-humans (such as environment in CSPP case) on the other.

When we rethink networked social activity, as Latour (2005) suggests, we must also reconsider the exclusivity of the human character of the social, as well as the fundamental structure of the action itself. That is, by platformization, social activity is now understood as an endeavor to represent a connecting type of action. As a result of digitalization, an ecology emerges that produces an ecosystem of relationships: unlike the outwardly projected social act, which is traditionally interpreted by sociology, the connecting act occurs within the network. In net-activism, action is not defined by an outside or an interior, nor by an object or a subject. There is no externalization of action in net-activism; rather, each action is an articulation of interactions between entities that come from a variety of relations.

A new paradigm of social action and involvement is evolving in both the PHM and CSP platforms, one that relies less on intermediate actors. For instance, platformization processes can transform institutional top-down decision-making procedures, increasing civic engagement by allowing citizens to actively participate in public administration or science (Kim, 2018). In contrast to hierarchical and closed organizations, net-activism argues for a collaborative, transparent, and accessible approach, with platforms serving as huge public places (Eysenbach, 2008).

As we have seen in the two case studies, platforms act as people’s representative places, engaging in a networked approach for the greater good and exchanging information and services with communities (Lupton, 2013; Helmond, 2015; Sharon and Lucivero, 2019). Informational factors are among the most effective motivators for people to prioritize their health, mostly in PHM platform.

If the first case study, the People’s Health Movement platform, involves social interaction among health associations, researchers, data, and people’s health movements, which are examples of info-medicine and prevention, as well as the foundation of a new dimension of public health social action, the second case study, the Citizen Sense Project platform, is also an example of crowdsourcing through citizen science. Platforms, as environments capable of accommodating various stakeholders, can lead to crowdsourcing, a process in which multiple intelligences (organic and inorganic) collaborate to solve problems by leveraging the capability of the digital system itself, such as big data or AI (Franzoni et al., 2022).

Crowdsourcing, or the process of bringing together non-experts and professionals to solve a problem, is becoming increasingly popular in public health because it allows various perspectives to define responses. This strategy can prevent ineffective top-down preventive interventions, save time and money, and expedite innovation. Crowdsourcing brings together top-down project management with bottom-up open innovation methodologies that were previously distinct in offline contexts (Brabham et al., 2014).

Our analysis of PHM and CSP platforms demonstrates how digital media can broaden the discussion of health practices beyond the traditional institutional connections and focus on preventive data entanglement. PHM and CSP platforms promote engagement methods that are at the crossroads of bio-digital-health citizenship and citizen science, owing to platform society system.

In the health contest, activism has evolved from the concept of battling for one’s rights to the endeavor to establish info-ecological profiles of action and attract new prospects. The PHM and CSP platforms can be used to carry out the type of action that Petersen et al. (2018) also refer to as bio-digital-citizenship in which a new social emerges, which is inextricably linked to the new digital tools, and which generates a new partnership with datafication (the so-called platform society). We live in a new common that is composed of data as well as physical realities; this is a world of bio-data-info-realities that are interconnected and interact with one another. People are now confronted with a new way of accessing health information in which various stakeholders are involved and data plays an important role. Such a procedure allows for better social accountability in a variety of institutional contexts, ranging from ordinary life to science (Dutton, 2023).

Actor theory defines action as a situation caused solely by the human subject’s will, which is directed externally. In a platform architecture, no one functions alone or in a transitive manner. Action occurs both outside and inside the context, serving a connective function, as evidenced by the two platforms analysis. This throws into question the fundamental nature of the social. Net-activism, as seen in PHM and CSP platforms, is the product of numerous agencies collaborating rather than a single subject. Platform action is a form-forming process that no entity can perform alone. Net-activism, like any biological system, manifests itself as an ecosystem comprised of both human and non-human agencies, in this sense is a trans-organic experience. We are witnessing a society that is more than just anthropocentric, including elements of culture, nature, data, and technologies.

## 6 Final considerations

Digital technologies provide new forms of interaction between humans and non-human agencies. We presented a more-than-human version of the social act based on our research into health platforms and platformization processes in a connected culture. This state of dependence between organic and inorganic agencies is creating an unbreakable network of biological and informational combinations as a result of digitalization processes. Specifically, as highlighted through platform analysis, it is a combination of people, data, and environmental factors through which we aimed to redefine social act as trans-organic. According to this viewpoint, a new living environment based on connection creates new frameworks for shared accountability among numerous stakeholders (not only humans), as well as communal decision-making (big data are part of the decision process). We live a new social that is both biological, cultural, and digital. Social networks are becoming trans-organic networks composed of people, big data, and other organic entities. We live in a new common that combines physical and informational realities.

A novel kind of ecology known as info-ecology is taking place, which is simultaneously biological and computational. According to the info-ecology perspective, the conventional separation between man and the environment, technology, and nature is replaced by an ecosystemic experience of a living state based on interconnections. Similarly, today’s promotion care pathways can be identified as info-ecosystems populated by numerous and differentiated channels (sensors, social networks, algorithms, data, peer relationships), which are altering the traditional rituals of face-to-face care that are typical of institutional interaction, including aftercare.

The case studies we presented demonstrate that the benefits of digital media extend beyond emotional and social support for people through online health forums. Platforms offer advantages such as co-creation and sharing of knowledge and health information, monitoring of urban quality of life, and population empowerment for individual and public health. Both platforms demonstrate how social action and health prevention are being reimagined as a connecting act on the one hand, and knowledge and data sharing on the other.

We referred to these networked engagement experiences as net-activism. The platforms under consideration are examples of net-activism via the lens of the info-ecological paradigm, which reimagines social action as collaborative, interconnected, and collective. That is, platforms are viewed as seeking to depict a networked act of a connected character that, by constructing an ecosystem of contacts, can differ from the traditional social act.

In contrast to the outwardly projected social act, also known as the traditional social act, networked action occurs on the network and, more crucially, with the network, and does not involve an outside or an interior, an object or a subject. There is no externalization of action in net-activism; rather, each act is an articulation of relations between entities. It is also important to remember that no entity in a network can act alone.

Digital media not only gives access to an infinite amount of information, in this case health-related information, but it also alters how people create and utilize that same information. The innovative dimension involves the fact that the new digital citizen is also a creator of content. On platforms, the subject can both collaborate and oppose other stakeholders, who are not always patients or people concerned about their own health, but can also include pharmaceutical firms, doctors, governments, and algorithms. Platforms replace hierarchical and closed structures with multi-sectoral collaborations, openness, democratic access to data, and public empowerment, all of which can be presented as examples of net-activism.

For those who use platforms to promote health net-activism, the struggle for individual rights is mixed with a desire to work together for various goals, such as attracting funds, advocating for public quality of life, influencing health legislation, or determining the best treatment. We established a conceptual framework that allows us to view health digital platforms as a complex ecology with a distinct communicative mode of life.

## Data Availability

The original contributions presented in the study are included in the article and are publicly available online, further inquiries can be directed to the corresponding author.
